# Short-Term Outcomes of Expedited Arthroscopic Tensionable Knotless Biologic Tuberoplasty for Massive Irreparable Rotator Cuff Tears

**DOI:** 10.31486/toj.23.0063

**Published:** 2023

**Authors:** Misty Suri, Arjun Verma, Sarena Matriano Lim, Justin Kim, Gregory Parker, Payton Baum, Jordan Nester

**Affiliations:** ^1^ Ochsner Andrews Sports Medicine Institute, Ochsner Clinic Foundation, Jefferson, LA; ^2^ Tulane University School of Medicine, New Orleans, LA; ^3^ Carle Illinois College of Medicine, University of Illinois at Urbana-Champaign, Urbana, IL

**Keywords:** *Arthroscopy*, *orthopedics*, *rotator cuff*, *rotator cuff injuries*

## Abstract

**Background:** Massive irreparable rotator cuff tears in the nonarthritic patient are challenging because of the high failure rate and technical difficulty of intraoperative repair. We examined the outcomes of expedited arthroscopic tensionable knotless biologic tuberoplasty for massive irreparable rotator cuff tears.

**Methods:** Eleven patients with an average follow-up of 8.2 months were included in the analysis. Patient-reported outcome measures were the visual analog scale (VAS) pain score, American Shoulder and Elbow Surgeons Standardized Shoulder Assessment Form (ASES) score, Single Assessment Numeric Evaluation (SANE) score, and Veterans RAND 12-Item Health Survey (VR-12) physical component score and mental component score.

**Results:** In comparison to the preoperative mean, mean VAS pain scores were significantly reduced at 2 weeks, 6 weeks, 3 months, 6 months, and 1 year. The mean VAS pain scores decreased from 6.9 ± 1.3 preoperatively to 0.2 ± 0.4 at 1 year (*P*<0.001). Mean ASES scores and SANE scores were both significantly improved at 3 months, 6 months, and 1 year. Mean ASES scores increased from 40.3 ± 17 preoperatively to 93.0 ± 5.5 at 1 year (*P*=0.001), and mean SANE scores increased from 40.7 ± 23.7 preoperatively to 85.6 ± 8.9 at 1 year (*P*=0.007). The mean VR-12 physical component score was significantly improved at 6 months and 1 year postoperatively. The mean VR-12 mental component score was clinically improved at 6 months and 1 year postoperatively.

**Conclusion:** Arthroscopic tensionable knotless biologic tuberoplasty is an effective treatment for massive irreparable rotator cuff tears and resulted in statistically significant improvements in VAS pain, ASES, SANE, and the VR-12 physical component scores and clinically significant improvements in the VR-12 mental component score in our patient cohort.

## INTRODUCTION

Massive irreparable rotator cuff tears in nonarthritic patients pose a unique challenge for orthopedic surgeons, as such tears present with intraoperative pathology that makes the creation of a stable repair construct difficult and technically demanding. While multiple operative techniques have been introduced, significant controversy remains over the optimal treatment. Various techniques include debridement with biceps tenotomy or tenodesis,^1,2^ various tendon transfer procedures,^3-5^ superior capsular reconstruction,^6^ biceps tendon rerouting,^7^ bursal acromial reconstruction,^8^ balloon spacers,^9^ and tuberoplasty.^10,11^

In 2002, Fenlin et al introduced the tuberoplasty technique for treating massive irreparable rotator cuff tears in patients who were not clinically suitable for shoulder arthroplasty.^12^ In this technique, a smooth articulation is created between the greater tuberosity of the humerus and the acromion via reshaping and contouring of the tuberosities, thereby allowing for articulation with the inferior aspect of the acromion and coracoacromial arch. With a mean follow-up of 27 months, patients reported significant reductions in pain and improvements in muscle strength.^12^

In 2004, Scheibel et al first described arthroscopic tuberoplasty using the term *reversed arthroscopic subacromial decompression*.^13^ In their study, reversed arthroscopic subacromial decompression, including tuberoplasty, provided satisfactory to excellent results at a mean follow-up of 40 months. Mirzayan et al further modified this technique after observing that in failed superior capsular reconstructions, clinical outcomes were still successful when the greater tuberosity remained covered by dermal allograft.^14^ Subsequently, Mirzayan and Bouz formally introduced the biologic tuberoplasty with acellular dermal allograft.^11^ In this technique, an acellular dermal allograft is used to cover the tuberosity, acting as a bumper and thereby preventing bone-on-bone contact between the tuberosity and the acromion. Suri et al further modified the technique to make it technically less demanding.^15^

For this investigation, we examined the postoperative outcomes of a technically expedited arthroscopic tensionable knotless biologic tuberoplasty technique for massive irreparable rotator cuff tears in nonarthritic shoulders.^15^ We present a modification of the Mirzayan and Bouz technique^11^ in which 4 total anchors, as opposed to 6, are used to secure the dermal allograft. We hypothesized that the patient cohort would illustrate statistically or clinically significant improvements in several validated patient-reported outcome measures in comparison to their preoperative scoring. We also hypothesized that patients would have an improved range of motion postoperatively.

## METHODS

This investigation was an institutional review board–approved retrospective review of 11 patients who underwent expedited arthroscopic biologic tuberoplasty for massive irreparable rotator cuff tears in nonarthritic shoulders.

### Patient Selection

Between December 2021 and March 2023, included patients underwent surgical treatment performed by the lead author (MS), a fellowship-trained sports medicine and shoulder reconstruction orthopedic surgeon in a high-volume shoulder practice. Inclusion criteria for this investigation were patients who underwent arthroscopic biologic tuberoplasty and had a minimum of 6 weeks of clinical follow-up and preserved function of the subscapularis muscle. Exclusion criteria included pseudoparalysis, infection, and advanced glenohumeral arthritis.

### Operative Technique

All operations were performed by the lead author with patients in the lateral position. A diagnostic arthroscopy is performed using standard posterior and anterior portals. The lateral portal is established, and the subacromial space is entered. A subacromial decompression is performed as needed, preserving the coracoacromial ligament. The rotator cuff is then examined arthroscopically and deemed irreparable, thereby being eligible for a biologic tuberoplasty. Concomitant procedures, such as biceps tenodesis and distal clavicle excision, are also performed as clinically appropriate.

The area to be covered by the graft is measured in the anterior-posterior and medial-lateral directions using an arthroscopic ruler.^15^ The 3-mm thick ArthroFLEX acellular dermal allograft (LifeNet Health) is prepped by an assistant on the back table. An arthroscopic burr is used to prepare a bleeding bone bed with gentle decortication on the greater tuberosity. Anterior and posterior 2.6-mm self-punching knotless FiberTak anchors (Arthrex, Inc) are percutaneously introduced through separate percutaneous portals just off the edge of the acromion and placed medially just off the articular margin. Anterior and posterior repair stitches are passed through the graft using the FiberLink sutures (Arthrex, Inc) previously placed during graft preparation. The repair stitches are shuttled through the medial anchors using the looped stitch of the tensionable knotless mechanism.^15^ The repair stitches are used to pull/shuttle the graft into place adjacent to the articular margin.^15^

**Figure 1.**
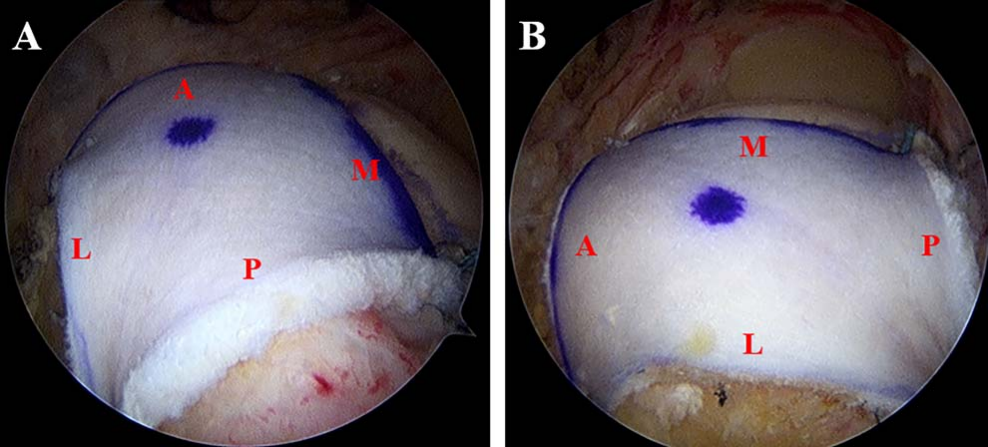
**(A) Posterior and (B) lateral views of a completed left shoulder arthroscopic tensionable knotless biologic tuberoplasty.** A, anterior; L, lateral; M, medial; P, posterior.

Once the graft has been shuttled into place with a ratcheting-type alternating tensioning of the anterior and posterior repair stitches, the lateral repair stitches are loaded onto anterolateral and posterolateral 4.75-mm SwiveLock anchors (Arthrex, Inc).^15^ After the graft is secured in place, appropriate tension and graft position are confirmed from both posterior and lateral viewing portals, completing the arthroscopic tensionable knotless biologic tuberoplasty (Figure 1).

### Postoperative Rehabilitation

The patient is placed in a standard abduction sling for 6 weeks postoperatively. Physical therapy starts at 7 to 10 days postoperatively and is initially geared toward pain reduction and passive range of motion with pendulum Codman exercises and periscapular function and activation. We allow weight-bearing on the operative arm as tolerated and allow active-assisted/active range of motion as tolerated, with strengthening to begin at 6 weeks postoperatively. The goal is pain-free range of motion by 3 months.

### Clinical Evaluations

All patients underwent a physical examination 1 to 3 days before the operation. Outpatient postoperative evaluations were performed regularly, and we recorded the results of the evaluations at 2 weeks, 6 weeks, 3 months, 6 months, and 1 year. Retrospective data collection was completed between September 2022 and April 2023. Demographic data recorded were age, sex, and body mass index.

Clinical outcome measures were the visual analog scale (VAS) pain score, American Shoulder and Elbow Surgeons Standardized Shoulder Assessment Form (ASES) score, Single Assessment Numeric Evaluation (SANE) score, and Veterans RAND 12-Item Health Survey (VR-12) physical component score and mental component score.

VAS pain is scored on a scale of 0 to 10, with 0 indicative of no pain and 10 indicative of maximal pain. The VAS pain score has a minimum clinically important difference (MCID) of 2.4 for arthroscopic rotator cuff repair.^16^ The ASES score is scored on a scale of 0 to 100, with higher scores indicative of better postoperative outcomes, and the ASES score has an MCID of 27.1 for arthroscopic rotator cuff repair.^16,17^ The SANE score is scored on a scale of 0 to 100, with higher scores indicative of better postoperative outcomes and has a MCID of 11.8.^18^ The VR-12 physical and mental component scores each have an average score of 50 and an MCID of 4.94 and 5.99, respectively.^19^ The VR-12 was developed from the Veterans RAND 36-Item Health Survey which was developed from the Medical Outcome Study RAND SF-36 Version 1.0.^20^ Shoulder range of motion measurements, including forward elevation, internal rotation, and external rotation, were also measured and recorded by the lead author.

Patients with massive irreparable rotator cuff tears were identified using preoperative magnetic resonance imaging (MRI) findings. Identification criteria included rotator cuff tears that were full-thickness tears of at least 2 tendons or measured ≥5 cm in diameter and tears presenting with torn tendon with retraction to the glenoid, Goutallier classification grade 3 to 4, acromiohumeral interval <6 mm, U-shaped tear, positive tangent sign on MRI, or superior migration of the humeral head.^21-27^

Full-thickness tears of at least 2 tendons, tears with retraction to the glenoid, Hamada grade 1, positive tangent sign, and Goutallier classification grade 3 to 4 were observed in our patients.

The Hamada classification system is used to characterize the stages of massive rotator cuff tears as they progress to rotator cuff arthropathy.^28^ Hamada grades range from 1 to 5, with 1 indicating an acromiohumeral interval >6 mm and 5 indicating humeral head collapse.^28,29^ Therefore, the severity of rotator cuff pathology/arthropathy increases with higher grades. The acromiohumeral interval is the distance between the inferior aspect of the acromion and the humeral head and is often used as a radiographic sign of rotator cuff tears.^29^ The acromiohumeral interval has been shown to be significantly negatively correlated with the size of rotator cuff tears.^30^ All patients included in this study were Hamada grade 1 with preserved joint space.

The tangent sign is an MRI finding in which the supraspinatus muscle does not cross a line extending from the superior border of the scapular spine to the superior border of the coracoid process.^31^ The tangent sign has been shown to be significantly associated with muscular atrophy and fatty infiltration of the supraspinatus tendon and thus is a highly sensitive and reliable identifier of irreparable rotator cuff tears on MRI.^25^

The Goutallier classification system characterizes the degree of fatty infiltration in the rotator cuff muscles via the following grades: 0 (muscle only), 1 (muscle predominates, limited fatty infiltration), 2 (increased fatty infiltration but muscle predominates), 3 (fatty infiltration and muscle in equal amounts), and 4 (fatty tissue exceeds muscle).^32^ Multiple investigations have demonstrated that higher amounts of preoperative fatty infiltration, as signified by higher Goutallier grades, are associated with poorer postoperative functional outcomes.^33,34^ Moreover, Goutallier grades 2 to 4 have been shown to be associated with a significantly higher re-tear rate than Goutallier grades 0 to 1.^35^

### Statistical Analysis

We queried the Surgical Outcomes System (SOS) database (Arthrex, Inc) to collect the functional outcome scores for patients who had undergone arthroscopic biologic tuberoplasty with the lead author. The SOS database is used to collect patient-reported outcome measures and send patients follow-up questionnaires. After querying the SOS database, we created a secure database of deidentified patient information using Microsoft Excel version 2211 (Microsoft Corporation). Descriptive statistics including frequencies, means, and standard deviations were calculated for all outcome measures. Statistically significant differences between preoperative and postoperative patient-reported outcome measures were identified using paired-samples *t* tests using R version 2022.07.1+554 (The R Foundation), with α=0.05.

## RESULTS

The 11 patients—8 males and 3 females—included in this investigation had a minimum of 6 weeks of clinical follow-up, and the average follow-up for this cohort was 8.2 months. The mean patient age was 65.6 ± 7.7 years, and the mean body mass index was 30.1 ± 4.1 kg/m^2^ (Table 1).

**Table 1.** Patient Demographics and Perioperative Details, n=11

Variable	Value
Sex	
Male	8 (72.7)
Female	3 (27.3)
Age, years, mean ± SD	65.6 ± 7.7
Body mass index, mean ± SD, kg/m^2^	30.1 ± 4.1
Tangent sign	
Yes	3 (27.3)
No	8 (72.7)
Tear dimensions, mm, mean ± SD	
Anterior-posterior	55.4 ± 12.7
Medial-lateral	47.5 ± 5.9
Goutallier classification grade	
1	6 (54.5)
2	2 (18.2)
3	2 (18.2)
4	1 (9.1)
Acromiohumeral interval, mm, mean ± SD	9.9 ± 2.4
Hamada grade 1	11 (100%)

Note: Data are presented as n (%) unless otherwise indicated.

Patient-reported outcome measure scores at all time points are presented in Table 2.

**Table 2.** Patient-Reported Outcomes

	Time Point					
Outcome Measure (Minimum Clinically Important Difference)	Preoperative, n=11	2 Weeks, n=11	6 Weeks, n=11	3 Months, n=11	6 Months, n=9	1 Year, n=5
VAS pain (MCID=2.4)	6.9 ± 1.3	3.7 ± 2.8^a^	1.4 ± 1.7^a^	1.1 ± 1.8^a^	0.6 ± 0.7^a^	0.2 ± 0.4^a^
		*P*=0.001	*P*<0.001	*P*<0.001	*P*<0.001	*P*<0.001
ASES (MCID=27.1)	40.3 ± 17.0	NA	NA	76.5 ± 17.4^a^	86.2 ± 10.7^a^	93.0 ± 5.5^a^
				*P*<0.001	*P*<0.001	*P*=0.001
SANE (MCID=11.8)	40.7 ± 23.7	NA	NA	72.3 ± 18.2^a^	81.1 ± 10.2^a^	85.6 ± 8.9^a^
				*P*=0.003	*P*=0.002	*P*=0.007
VR-12 PCS (MCID=4.94)	33.7 ± 6.8	NA	NA	NA	44.0 ± 7.0^a^	49.7 ± 4.9^a^
					*P*=0.003	*P*=0.016
VR-12 MCS (MCID=5.99)	46.4 ± 12.2	NA	NA	NA	57.2 ± 10.6^a^	56.5 ± 12.5^a^
					*P*=0.053	*P*=0.252

^a^Values that surpassed the minimum clinically important difference and are thus clinically significant findings.

Notes: Data are presented as mean ± standard deviation. For all scores except VAS pain, higher scores are indicative of better outcomes. VAS pain is scored on a scale of 0 to 10, with 0 indicative of no pain and 10 indicative of maximal pain. The *P* values indicate the statistical significance compared to the respective preoperative score and were calculated from paired-samples *t* tests.

ASES, American Shoulder and Elbow Surgeons Standardized Shoulder Assessment Form; MCID, minimum clinically important difference; NA, not assessed (at the specified time point); SANE, Single Assessment Numeric Evaluation; VAS, visual analog scale; VR-12 MCS, Veterans RAND 12-Item Health Survey mental component score; VR-12 PCS, Veterans RAND 12-Item Health Survey physical component score.

**Figure 2.**
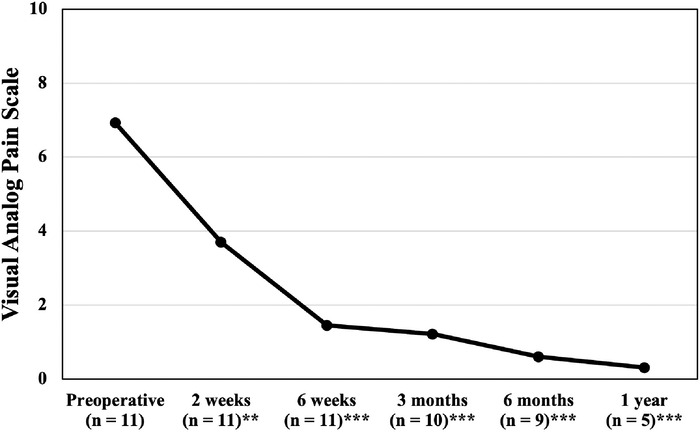
Mean visual analog scale (VAS) pain scores from preoperative to 1-year postoperative follow-up. VAS pain is scored on a scale of 0 to 10, with 0 indicative of no pain and 10 indicative of maximal pain. Statistically significant differences in comparison to the preoperative score are identified with ^**^ (*P*<0.01) and ^***^ (*P*<0.001).

In comparison to preoperative scores, this patient cohort exhibited clinically and statistically significant reductions in mean VAS pain scores at all postoperative follow-up time points: 2 weeks, 6 weeks, 3 months, 6 months, and 1 year. The mean VAS pain score was 6.9 ± 1.3 preoperatively, decreasing to 0.2 ± 0.4 at 1 year (*P*<0.001). VAS pain scores are graphed in Figure 2.

**Figure 3.**
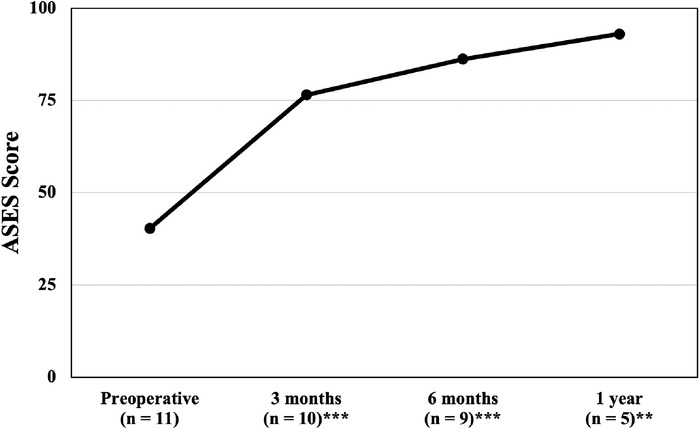
Mean American Shoulder and Elbow Surgeons Standardized Shoulder Assessment Form (ASES) scores from preoperative to 1-year postoperative follow-up. The ASES is scored on a scale of 0 to 100, with higher scores indicative of better postoperative outcomes. Statistically significant differences in comparison to the preoperative score are identified with ^**^ (*P*<0.01) and ^***^ (*P*<0.001).

In comparison to preoperative scores, the increases in the mean ASES score were both clinically and statistically significant at all follow-up time points: 3 months, 6 months, and 1 year postoperatively. The mean preoperative ASES score was 40.3 ± 17.0, increasing to 93.0 ± 5.5 at 1 year postoperatively (*P*<0.001). ASES scores are graphed in Figure 3.

**Figure 4.**
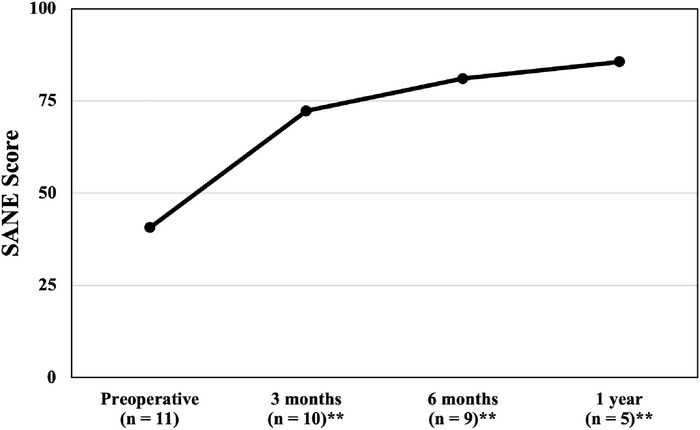
Mean Single Assessment Numeric Evaluation (SANE) scores from preoperative to 1-year postoperative follow-up. The SANE is scored on a scale of 0 to 100, with higher scores indicative of better postoperative outcomes. Statistically significant differences in comparison to the preoperative score are identified with ^**^ (*P*<0.01).

The differences between preoperative and postoperative SANE scores were statistically and clinically significant at all follow-up time points (3 months, 6 months, and 1 year postoperatively). The mean preoperative SANE score was 40.7 ± 23.7, increasing to 85.6 ± 8.9 at 1 year postoperatively (*P*=0.007). SANE scores are graphed in Figure 4.

**Figure 5.**
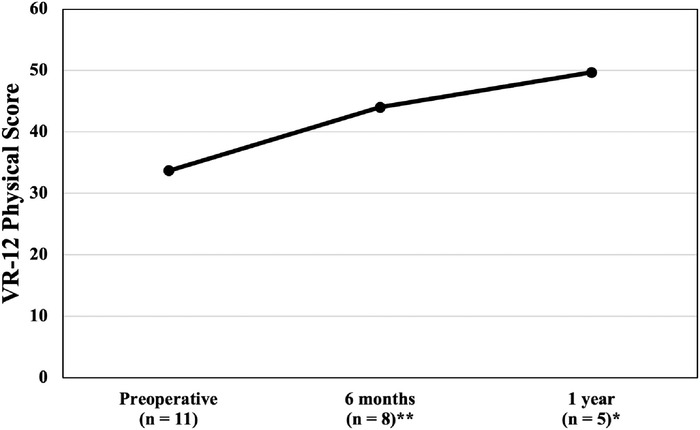
Veterans RAND 12-Item Health Survey (VR-12) physical component scores from preoperative to 1-year postoperative follow-up. Higher scores are indicative of better outcomes. Statistically significant differences in comparison to the preoperative score are identified with ^*^ (*P*<0.05) and ^**^ (*P*<0.01).

**Figure 6.**
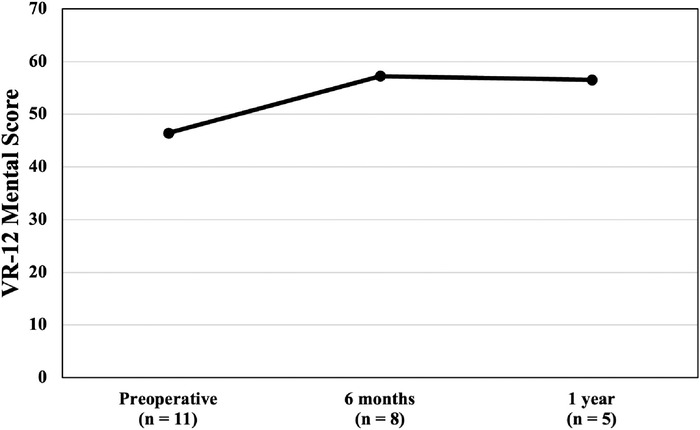
Veterans RAND 12-Item Health Survey (VR-12) mental component scores from preoperative to 1-year postoperative follow-up. Higher scores are indicative of better outcomes. The scores at the 2 postoperative timepoints were not statistically significantly different from the preoperative score.

The improvements in the VR-12 physical component score were clinically and statistically significant at both follow-up time points: 6 months and 1 year. The preoperative mean VR-12 physical component score was 33.7 ± 6.8, increasing to 49.7 ± 4.9 at 1 year postoperatively (*P*=0.016). The VR-12 physical component scores are graphed in Figure 5. The difference between the preoperative and postoperative VR-12 mental component scores was clinically significant at 6 months and 1 year. However, the improvements in the mean VR-12 mental component score were not statistically significant at either time point. The mean VR-12 mental component score increased from 46.4 ± 12.2 preoperatively to 57.2 ± 10.6 at 6 months and declined slightly to 56.5 ± 12.5 at 1 year postoperatively. The VR-12 mental component scores are graphed in Figure 6.

Range-of-motion measurements also improved in this patient cohort (Table 3). Mean forward elevation increased from 145.0 ± 37.2 preoperatively to 169.0 ± 7.3 at 1 year postoperatively. Compared to the preoperative mean of 37.8 ± 10.8, mean external rotation improved to 46.0 ± 2.0 at 1 year postoperatively. Mean internal rotation also improved from L3 preoperatively to L1 at 1 year postoperatively. The differences in range of motion were not significant at any follow-up time points.

**Table 3.** Average Range of Motion

	Time Point			
Variable	Preoperative, n=11	3 Months, n=11	6 Months, n=9	1 Year, n=5
Forward elevation, degrees	145.0 ± 37.2	153.0 ± 33.2	166.7 ± 9.4	169.0 ± 7.3
External rotation, degrees	37.8 ± 10.8	44.4 ± 10.1	45.0 ± 2.4	46.0 ± 2.0
Internal rotation^a^	L3	L2	L1	L1

^a^Internal rotation is measured by the maximal vertebral level reached by the patient's thumb.

Note: Data are presented as mean ± standard deviation.

## DISCUSSION

As already stated, various techniques are used to treat massive irreparable rotator cuff tears, including debridement with biceps tenotomy or tenodesis,^1,2^ various tendon transfer procedures,^3-5^ superior capsular reconstruction,^6^ biceps tendon rerouting,^7^ bursal acromion reconstruction,^8^ balloon spacers,^9^ and the tuberoplasty.^10,11^ Anterior deltoid strengthening exercises have been shown to result in improvements in Constant-Murley score and forward elevation in patients presenting with preoperative pseudoparalysis who were medically unfit for surgery.^36^

In the 2002 study in which Fenlin et al introduced the original tuberoplasty technique for treating massive irreparable rotator cuff tears in patients who were not clinically suitable for shoulder arthroplasty, 13 patients (68%) were completely pain-free after a mean 27-month follow-up, all patients were able to perform activities of daily living, and 9 of 11 who were employed preoperatively returned to work.^12^ Using a similar technique, Lee et al showed excellent results in their series of 32 patients who underwent arthroscopic tuberoplasty with mean follow-up of 40 months. The Constant-Murley score increased from a preoperative mean of 47.6 points to 70.4 points.^37^

In 2021, Mirzayan and Bouz introduced the arthroscopic biologic tuberoplasty technique using a limited acellular dermal allograft resurfacing of just the greater tuberosity.^11^ This technique provides an option for patients who are unwilling or unable to undergo shoulder replacement or more extensive arthroscopic operations because of medical comorbidities. This arthroscopic biologic tuberoplasty concept stemmed from investigation of previous failed dermal allograft procedures for irreparable rotator cuff tears. Mirzayan et al analyzed the results of 25 shoulders with massive rotator cuff tears that underwent a bridging procedure or superior capsular reconstruction utilizing acellular dermal matrices.^14^ Postoperative MRI was used to identify the status of the graft: type I (intact graft); type II (graft tear with tuberosity covered); and type III (graft tear with tuberosity uncovered [bare]). The Mirzayan et al analysis revealed statistically significant postoperative VAS score improvements from baseline: 7.0 vs 0.7 for type I grafts and 8.1 vs 1.3 for type II grafts. Importantly, no difference was found in VAS results between type I and type II grafts.^14^ This significant pain relief is also supported by our preliminary results, shown through a statistically significant reduction in VAS pain scores at all time points postoperatively.

The results of the Mirzayan et al analysis led Mirzayan and Bouz to introduce the arthroscopic biologic tuberoplasty technique, which entails a transosseous equivalent rotator cuff repair technique with 3 medial and 3 lateral row anchors with a crossing suture tape configuration.^11^ While effective, this technique requires a significant amount of suture management and may be technically challenging. The Suri et al technique is an expedited modification of this technique, using only 2 medial and 2 lateral anchors.^15^ We believe the Suri et al technique is reproducible and intraoperatively efficient and results in similar rates of graft healing and pain control compared to the Mirzayan and Bouz technique.

Our study has limitations. The number of included patients is small and the follow-up is relatively short. This technique is relatively new and was developed by the lead author of this investigation; therefore, the data are from 1 surgeon at 1 institution. However, as no study of the short-term outcomes of arthroscopic biologic tuberoplasty has been published, we feel these limitations are justified. Second, the retrospective nature of this study imposes limitations inherent to all retrospective studies, such as difficulty controlling bias or confounders. Other limitations include the lack of a control group and the absence of postoperative MRIs for our patients.

## CONCLUSION

The use of the expedited arthroscopic biologic tuberoplasty technique as a technically efficient treatment for massive irreparable rotator cuff tears resulted in improved clinical outcomes and pain reduction at all time points of follow-up (mean of 8.2 months) in our cohort of patients. As an alternative to arthroplasty or for patients who are poor candidates for arthroplasty or more extensive arthroscopic procedures, this technique is reproducible and technically efficient.

## Notes

This article meets the Accreditation Council for Graduate Medical Education and the American Board of Medical Specialties Maintenance of Certification competencies for Patient Care and Medical Knowledge.
